# A Hemiclamshell Incision for a Giant Solitary Fibrous Tumor of the Right Hemithorax

**DOI:** 10.1155/2012/826454

**Published:** 2012-11-07

**Authors:** Nilgün Kanlıoğlu Kuman, Serdar Şen, Salih Çokpınar, Emel Ceylan, Canten Tataroğlu, Mehmet Boğa

**Affiliations:** ^1^Department of Thoracic Surgery, Adnan Menderes University, 09000 Aydın, Turkey; ^2^Department of Pulmonology, Adnan Menderes University, 09000 Aydın, Turkey; ^3^Department of Pathology, Adnan Menderes University, 09000 Aydın, Turkey; ^4^Department of Cardiovascular Surgery, Adnan Menderes University, 09000 Aydın, Turkey

## Abstract

A 41-year-old female was admitted with respiratory distress. Chest radiographs showed opacity in the right hemithorax with mediastinal shift. Computed tomography (CT) scan showed a pleural mass with a 22 cm diameter occupying the whole right hemithorax and causing atelectasis. Magnetic resonance imaging (MRI) showed lower position of the right hemidiaphragm and the liver. Superior vena cava and heart were shifted to left. Presence of infiltration to the adjacent tissues could not be clearly evaluated because of pressure effect. Transthoracic needle biopsy specimen was reported to be benign. Because of the size and location of the mass, a hemiclamshell incision was chosen, which allowed excellent visualization and complete dissection of the giant tumor. The histopathology of the resected specimen confirmed solitary fibrous tumor. The patient was stabilized by careful observation and treatment. No complication except pneumonia in the postoperative first month occurred during the 22-month follow-up period.

## 1. Introduction

Incidence of solitary fibrous tumor of the pleura was reported as 2.8/100.000. Tumor originates from visceral pleura 80%, most are asymptomatic, and malignancy rate has been reported as 10–30%. Primary treatment of these tumors is surgery. Mediastinal or diaphragmatic pleural tumor, recurrent tumor originating from the parietal pleura, or tumors larger than 10 cm have malignancy potential and should be followed [1].

Symptom rate increases with tumor size, and common symptoms were cough, dyspnea, and chest pain. A well-defined homogeneous soft tissue mass with pleural basis with effusion occasionally was observed at radiological examination. CT and MRI are mandatory to determine the relationship of mass with surrounding tissues in huge tumors.

## 2. Case

**Figure 1:**
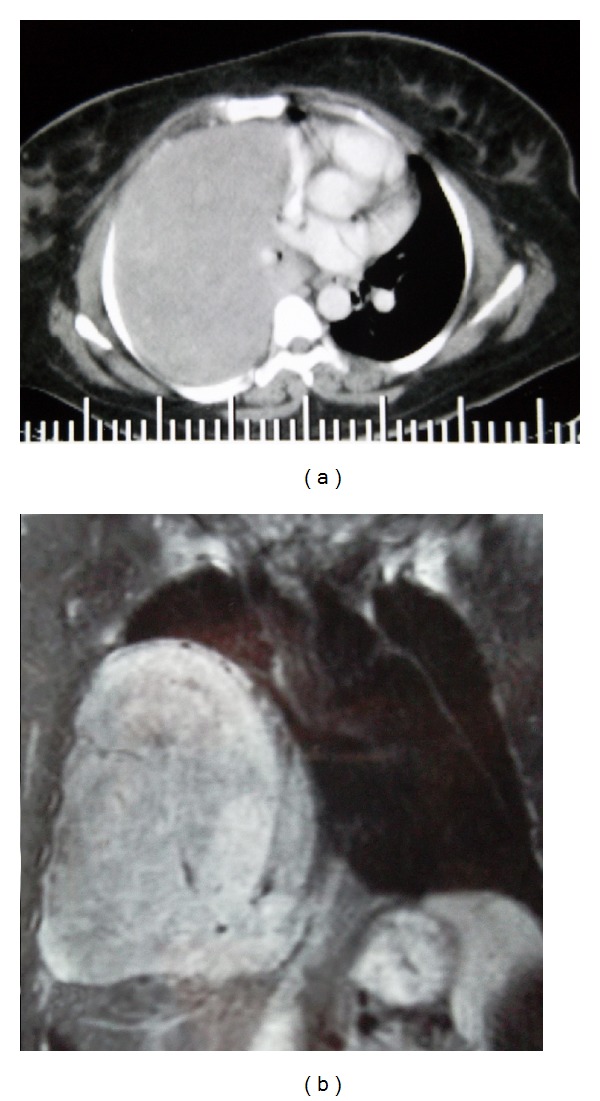
Giant mass can be seen that almost completely filled the right hemithorax on CT (a). Mediastinal shift was seen on MRI (b).

A 41-year-old female patient was admitted to the emergency department with shortness of breath, blunt chest pain, and fatigue. In examination of the respiratory system, there were no breath sounds in the right middle and lower zones. Abdominal distension, umbilical hernia, and edema of the lower limbs were present. She had multinodular goitre. In her medical history, there was a lung pathology for which surgery was recommended three years ago. Laboratory findings were CRP: 243 mg/L; pulmonary function tests: restrictive pulmonary dysfunction; arterial blood gas analyses: respiratory acidosis and hypercarbia; thyroid function tests: euthyroid. Opacity was seen in the right hemithorax extending from the anterior branch of the 2th rib to the diaphragm in radiological studies. Thorax CT showed a giant mass that almost completely filled the right hemithorax, right pleural effusion, and right lung atelectasis. A 20 cm diameter mass that filled the whole right hemithorax extending from the upper mediastinum to the left of the midline was seen in thoracic magnetic resonance imaging. Right hilus and heart were pushed to the the left, and there was no evidence of mediastinal invasion. Inferior vena cava and hepatic veins were enlarged, and congestion of the liver, splenomegaly, and multinodular goiter were reported (Figures 1(a) and 1(b)).

Abdominal ultrasonography revealed hepatosplenomegaly. Transthoracic biopsy was reported as benign mesenchymal tumor. Explorative thoracotomy revealed a huge extrapleural mass, and right upper lobe was adherent to the mass, and there were a large number of vascular structures. The relation of the mass with mediastinal structures and diaphragm and the vascular structures between the tumor and cardiac, mediastinal, and diaphragmatic surfaces could not be the evaluated through the thoracotomy incision. Hemorrhage developed during the separation of adhesions, and hemodynamics deteriorated because of the mass pressure on the heart. The existence of multiple fragile vascular structures on mediastinal and cardiac surfaces like those on the lung surface would make the control of the hemorrhage difficult and increase the mortality risk with hemodynamic problems. We thought that removal of the tumor and control of vascular structures with circulatory arrest could be more secure, if necessary. The operation was terminated to take a consult from cardiovascular surgery and to take approval from the patient because the resection was not safe through that incision.

**Figure 2:**
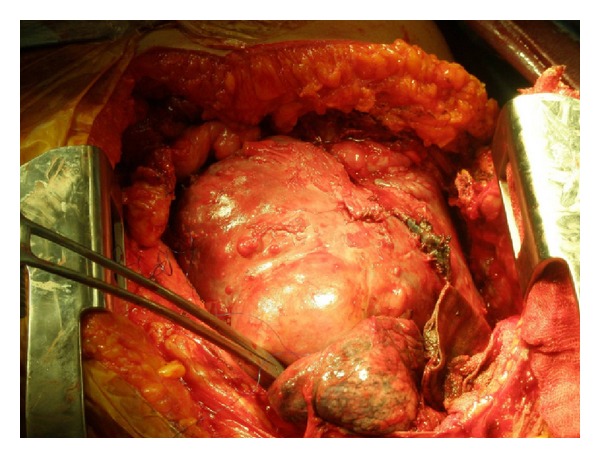
Solitary fibrous tumor which filled the entire hemithorax causing mediastinal shift.

In the second operation we were prepared for a possible need for deep hypothermic circulatory arrest by cardiopulmonary bypass to control hemorrhage. A hemiclamshell incision was performed in the second postoperative day for resection of the mass through anterolateral thoracotomy incision combined with median sternotomy (Figure 2).

**Figure 3:**
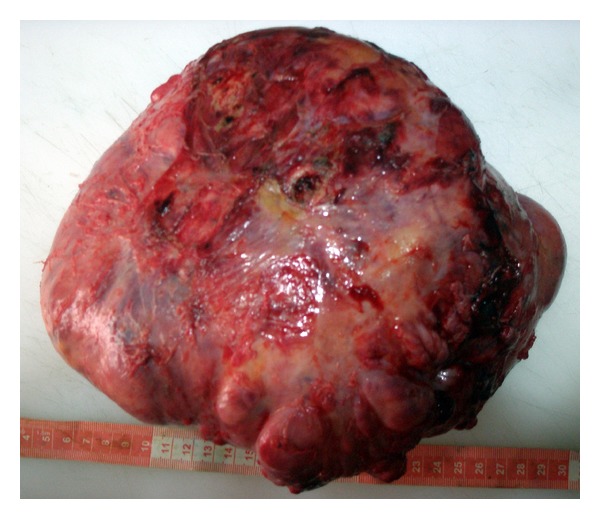
The tumor after excision.

Vascular structures originating from mediastinum and diaphragm were observed. The tumor was excised en bloc without the need for circulatory arrest. The tumor was 25 × 18 cm in size and 2590 gr in weight (Figure 3).

**Figure 4:**
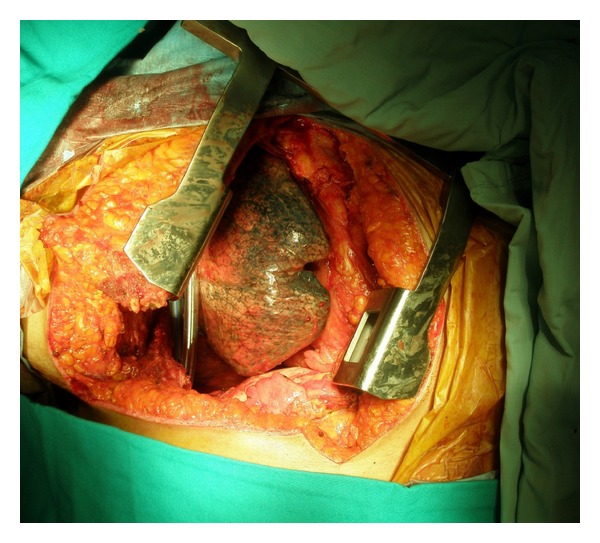
Hemiclamshell incision can be observed after removal of the tumor. A separator was placed on borders of the sternotomy incision.

The lower and middle lobe collapses were resolved with removal of the tumor. Two drains were placed into the thorax, and the incision was closed after local bupivacaine injection (Figure 4).

**Figure 5:**
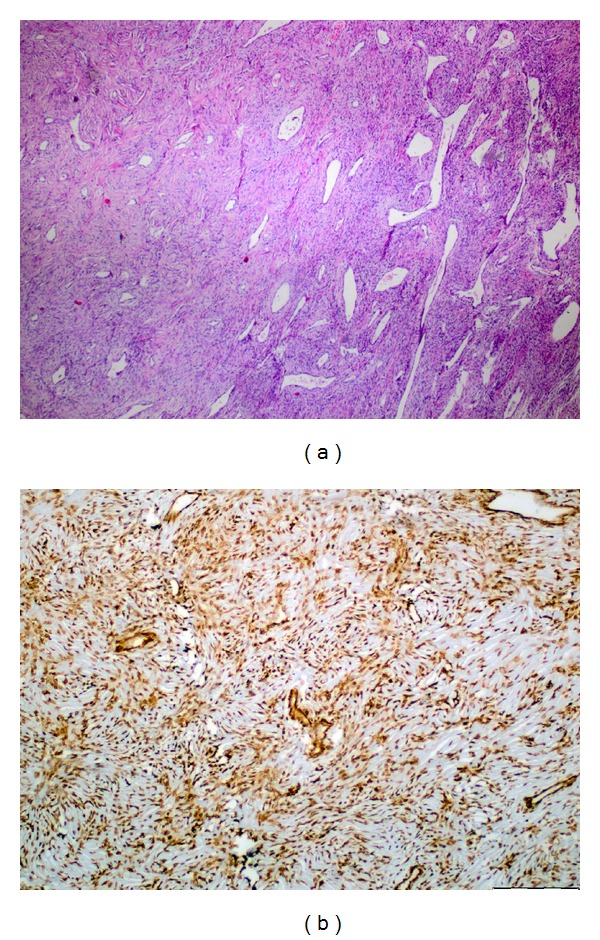
(a) Spindle-shaped cells with indistinct borders and branching hemangiopericytoma-like vessels (H & E, ×100). (b) Strong immuno-reactivity of the tumor cells for CD34 in solitary fibrous tumor (CD34, ×200).

The pathologic examination revealed that the tumor surface was covered with a single layer of mesothelial cells and underlying hypo- and hypercellular areas of spindle cells. The lesion had whorl structures, wave pattern, brightly eosinophilic collagen bundles, hemangiopericytomal vascular structures in the form of deer antler, and large areas of necrosis and calcification. One mitosis was observed in ten magnifications. In immunohistochemical examination, the tumor was stained with CD34 and actin strongly and diffuse, and with CD99, S100, Blc2 weakly. The surface mesothelial cells were stained with cytokeratin. With these findings the lesion was reported as a solitary fibrous tumor (Figure 5).

Analgesia was given via epidural catheter as tramadol infusion in the first postoperative day. The next day epidural catheter was terminated and tramadol combined with nonsteroid anti-inflammatory agents were given. Tramadol was stopped in 8th postoperative day and analgesia was continued with nonsteroid anti-inflammatory agents. Drains were removed on the 2th and 7th postoperative days. In the 4th postoperative day, atelectasis of the right lower lobe developed because of retention of secretions. With repetitive bronchial lavage and CPAP (continuous positive airway pressure) therapy, the clinical and radiologic findings were improved and the patient was discharged on the 11th postoperative day. The patient was internalized with pneumonia after 12 days. The clinical condition of patient was improved with mechanical ventilation and antibiotic treatment, and she was discharged on the 23th day of second internalization.

## 3. Discussion

Hemiclamshell incision is a combination of anterolateral thoracotomy and median sternotomy incision. This incision was usually used for vascular injuries in mediastinal or anterolateral cervicothoracic junction and resection of tumors located in the cervicothoracic area or extended to the anterior mediastinum [2].

Lateral decubitus position of conventional thoracotomy should not be preferred in large-sized tumor operations because the pressure of the tumor on the heart and decreased venous return result with deterioration of cardiac hemodynamics due to lateral decubitus position as in our case. The reason why we prefer thoracotomy in the first operation was to avoid a larger incision, explore the vascular connection between tumor, lung, and mediastinum, and try to excise it if possible. But the tumor had numerous vascular connections with lung and because of the position of the mass, vascular structures on cardiac, mediastinal, and diaphragmatic surfaces of the tumor could not be evaluated. Hemiclamshell incision provides manipulation of these tumors as well as protection of hemodynamics [3]. We did not continue the operation with hemiclamshell incision to provide hemodynamic improvement after hemorrhage. 

One of the advantages of the hemiclamshell incision was to provide the optimal exposure of hilar and mediastinal vascular structures. Vascular structures are better observed, and possible vascular injury could be more easily manipulated through hemiclamshell incision. In cases that mediastinal, cardiac vascular structures, and feeding vessels of the mass cannot be properly evaluated due to tumor, control of vascular structures after removal of the tumor with circulatory arrest could be more secure according to our opinion. A multidisciplinary approach will be helpful in the management of tumors like this one. Hemiclamshell incision provides shoulder stability and sternoclavicular joint function protection compared with previously described incisions for the anterior cervicothoracic region tumors in addition [4]. In tumor operations, this incision may provide access to many mediastinal lymph node stations and lymph node sampling. 

Pain and atelectasis were reported as the most common complications of hemiclamshell incision. We gave adequate analgesia to the patient, and she did not suffer from pain before or after discharge, but she had long-term compression atelectasis, which could have been predisposed to pneumonia. High preoperative CRP level may be indicative of subclinical pulmonary infection that became visible after surgery. In our case mucus retention and resistance to pulmonary rehabilitation might be considered as causes of pneumonia after discharge. The study of Lardinois et al. revealed no significant difference in the incidence of complications after hemiclamshell incision compared with conventional incisions [2].

In conclusion, the hemiclamshell incision provides optimal exposure and manipulation for the resection of intrathoracic giant tumors. Complications may be seen due to the underlying disease or injury, and there is no difference in complication rates compared with conventional incisions. To reduce complications, patient compliance is important as far as postoperative care like all thoracic incisions.
